# Diagnostic Performance of 3.0-Tesla Magnetic Resonance Imaging for Pulmonary Nodules and Masses: A Comparative Cross-Sectional Study With Multidetector Computed Tomography

**DOI:** 10.7759/cureus.111727

**Published:** 2026-06-29

**Authors:** Tamphasana Maimom, Longjam Ronibala, Kavitha B Gowda, Pooja Sharma, Soibam Subhaschandra, Sunanda Haorongbam

**Affiliations:** 1 Radiodiagnosis, Regional Institute of Medical Sciences, Imphal, IND; 2 Radiology, Tenet Diagnostics Centre, Bengaluru, IND; 3 Chest Medicine, Regional Institute of Medical Sciences, Imphal, IND

**Keywords:** 3-tesla mri, lung mass, lung mri, mdct, nodule detection, pulmonary nodule, radiation-free imaging

## Abstract

Background

Pulmonary nodules are increasingly detected incidentally on computed tomography (CT) scans, requiring serial follow-up examinations that carry a non-trivial cumulative radiation burden. Magnetic resonance imaging (MRI) at 3.0 Tesla (3T) offers comparable soft-tissue contrast without ionizing radiation and may represent a viable alternative for nodule surveillance. This study evaluated the diagnostic performance of 3T MRI for detecting pulmonary nodules and masses in comparison with multidetector computed tomography (MDCT), which served as the reference standard.

Methodology

This hospital-based, cross-sectional study enrolled 60 adult patients with CT-confirmed pulmonary nodules or masses at the Department of Radiodiagnosis. All participants underwent 3T MRI (Siemens Skyra) using multiple non-contrast sequences: half-Fourier acquisition single-shot turbo spin-echo (T2-HASTE), volumetric interpolated breath-hold examination (T1-VIBE), true fast imaging with steady-state free precession (T2-TRUFI), T2-BLADE (periodically rotated overlapping parallel lines with enhanced reconstruction), and echo planar imaging in two dimensions diffusion-weighted imaging (Ep2d DWI). Lesion number, size, location, margins, cavitation, pleural effusion, and mediastinal lymphadenopathy were recorded. Overall and size-stratified sensitivities of MRI were calculated with MDCT as a reference. Bland-Altman analysis assessed measurement agreement between the two modalities.

Results

MDCT identified 108 nodules or masses in 60 patients (mean age = 49.67 ± 17.80 years; female:male ratio = 1.14:1). MRI detected 94 lesions (overall sensitivity 87%). Size-stratified sensitivity was 9% for nodules <4 mm, 75% for 4-5 mm, 95.2% for 5-7 mm, and 100% for >7 mm. Bland-Altman analysis showed excellent size agreement (mean bias = 0.65 mm; limits of agreement = −1.5 to +2.5 mm). Pleural effusion, mediastinal lymphadenopathy, and cavitation were concordantly identified on both modalities.

Conclusions

3T MRI demonstrated high sensitivity for pulmonary nodules exceeding 7 mm and excellent size-measurement agreement with MDCT. Given its freedom from ionizing radiation and reliable performance for clinically significant nodules, 3T MRI is a promising radiation-free alternative for surveillance of pulmonary nodules in appropriate clinical contexts, particularly for patients requiring repeated imaging.

## Introduction

Pulmonary nodules, defined as approximately rounded opacities measuring up to 3 cm in maximum diameter, represent one of the most common incidental findings on thoracic imaging. Lesions exceeding 3 cm are conventionally classified as pulmonary masses. With the widespread adoption of multidetector computed tomography (MDCT) for a variety of thoracic indications and, more recently, for low-dose lung cancer screening in high-risk populations, the frequency with which nodules are detected has risen substantially [1,2]. National lung cancer screening trials demonstrated that low-dose computed tomography (CT) screening is associated with a significant reduction in lung cancer mortality; however, the ensuing challenge lies in managing the large number of screen-detected nodules that prove benign on follow-up [3].

Current guidelines recommend serial CT imaging at defined intervals to assess nodule growth and estimate malignant potential. This approach imposes a cumulative radiation burden that is not inconsequential, particularly in younger patients and those undergoing long-term oncological surveillance. Brenner and colleagues estimated that annual CT screening could increase the absolute risk of radiation-induced lung cancer by nearly 0.85% in a 50-year-old female smoker over a 25-year screening period [4]. These concerns have motivated considerable interest in imaging alternatives that preserve diagnostic performance while eliminating ionizing radiation exposure.

Magnetic resonance imaging (MRI) of the lung has historically been constrained by the inherently low proton density of aerated lung parenchyma, susceptibility to respiratory and cardiac motion artifacts, and rapid signal decay associated with short T2* relaxation times. However, technical advances over the past decade have substantially improved the feasibility of pulmonary MRI. High-performance gradient systems, multi-channel phased-array receiver coils, parallel imaging techniques, and optimized sequences with ultrashort echo times (UTEs) have collectively increased the signal-to-noise ratio and spatial resolution achievable in pulmonary examinations [5,6]. The transition to 3.0-Tesla (3T) field strength provides an inherent doubling of signal-to-noise ratio compared with 1.5T systems, a benefit that can be translated into improved spatial resolution or shorter acquisition times [7].

A growing body of literature supports the use of MRI for pulmonary nodule detection, with reported sensitivities ranging from 48% to 100% depending on nodule size, the MRI sequence employed, and the field strength used [8-11]. Most studies have reported excellent performance for lesions larger than 6-8 mm, with sensitivity declining substantially for sub-5 mm nodules [12,13]. Nevertheless, the majority of clinically actionable nodules, those warranting active surveillance or biopsy, fall within the size range where MRI is reported to perform adequately.

Despite this encouraging evidence, data from 3.0T systems examining a broad spectrum of pulmonary nodule sizes, including multiple complementary sequences, remain limited in Indian patient cohorts. The present study was therefore designed to prospectively evaluate the diagnostic sensitivity of a multi-sequence 3T MRI protocol for detecting pulmonary nodules and masses and to assess the accuracy of MRI-based size measurement relative to MDCT.

## Materials and methods

Study design and setting

This was a prospective, hospital-based, cross-sectional study conducted in the Department of Radiodiagnosis, Regional Institute of Medical Sciences (RIMS), Imphal, Manipur, India, in collaboration with the Department of Respiratory Medicine. Ethical approval was obtained from the Institutional Ethics Board (A/206/REB-Comm (SP)/RIMS/2015/868/206/2022). Written informed consent was obtained from all participants. The study was conducted from May 2022 to June 2024.

Study population

A total of 60 adult patients (aged ≥18 years) with one or more pulmonary nodules or masses identified on prior MDCT were consecutively enrolled from the Departments of Respiratory Medicine and Radiodiagnosis. Inclusion criteria included the willingness and physical ability of patients to undergo MRI. Exclusion criteria included standard contraindications to MRI (presence of a cardiac pacemaker, ferromagnetic metallic implants, or severe claustrophobia) and the inability to comply with breath-hold instructions due to active respiratory irregularities or dyspnea.

The minimum required sample size was calculated using the following formula: n = Z²α/2 × Sn × (1−Sn)​/d²

Where: Sn = expected sensitivity = 80.5% (0.805), as reported by Cieszanowski et al. [14]; Zα/2 = 1.96 (for 95% confidence level); and d = margin of error = 10% of 80.5% = 0.0805.

Accordingly, n = (1.96)² × 0.805 × (1−0.805)/(0.0805)² = 3.8416 × 0.805 × 0.195/0.006480 ≈ 47

Adding a 20% dropout allowance to account for patient non-compliance, MRI contraindications identified after enrollment, and technically inadequate examinations, the minimum sample size was inflated to 60 participants. A total of 60 patients were successfully recruited and completed both MDCT and MRI examinations, with no attrition recorded during the study period.

Imaging equipment

**Figure 1:**
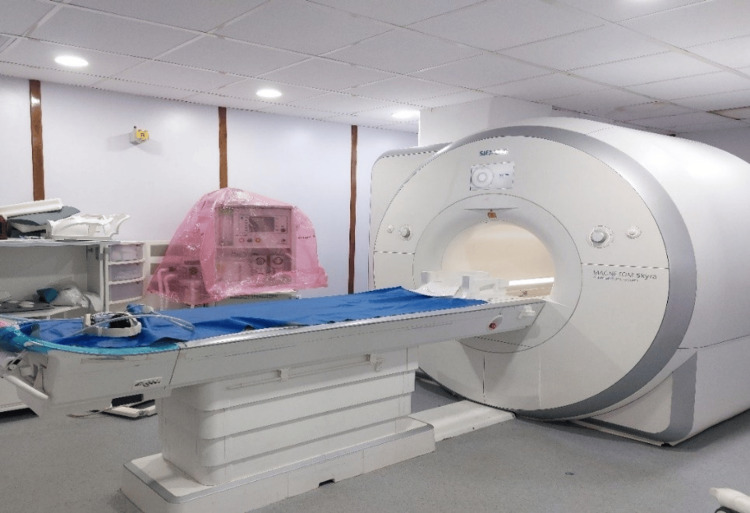
Siemens 3.0-Tesla MRI scanner (Skyra, Erlangen, Germany) used for all MRI examinations in this study. The scanner is equipped with Explorer gradients (maximum gradient strength = 45 mT/m; slew rate = 200 mT/m/ms) and a phased-array multi-channel body coil. MRI = magnetic resonance imaging

Two imaging platforms were used throughout the study period. MRI examinations were performed on a 3.0T Siemens Skyra scanner (Erlangen, Germany) equipped with a phased-array multi-channel body coil (Figure 1).

**Figure 2:**
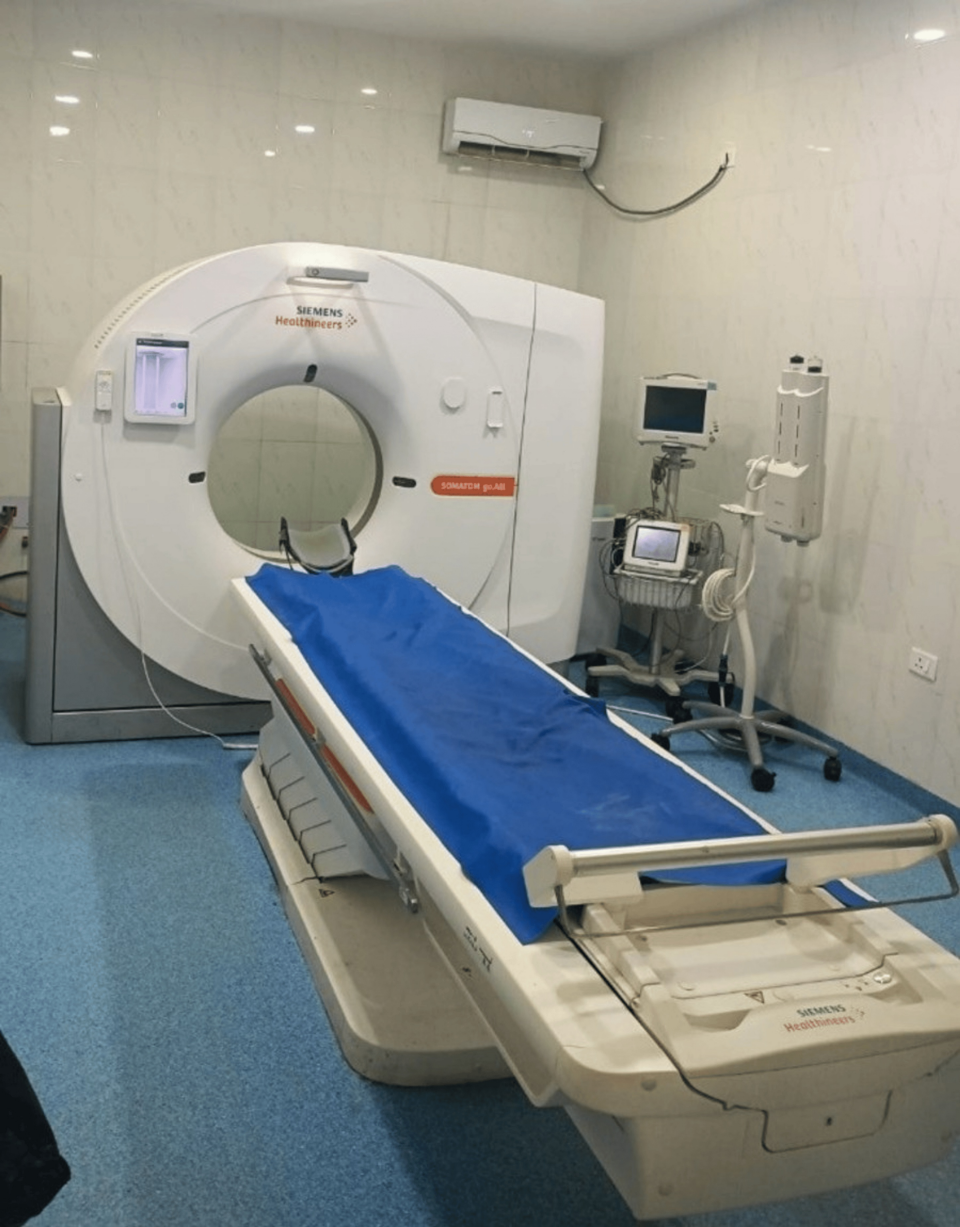
Siemens SOMATOM Go ALL 64-slice multidetector CT scanner used as the reference standard imaging platform. Acquisition parameters: 1.25 mm collimation, 120 kVp, 120 mAs; reconstructed in axial and coronal planes at 3 mm slice thickness. CT = computed tomography

CT examinations were performed on a Siemens SOMATOM Go ALL 64-slice scanner (Siemens Healthineers, Erlangen, Germany) (Figure 2). Both scanners were housed within the Department of Radiodiagnosis.

Computed tomography imaging protocol

Non-contrast MDCT was performed using a 64-detector-row Siemens SOMATOM Go ALL scanner (Siemens Healthineers, Erlangen, Germany) with the following parameters: detector collimation, 1.25 mm; table feed, 0.938-0.984 mm per rotation; tube voltage, 120 kVp; and tube current, 120 mAs. Images were reconstructed in axial and coronal planes at a 3 mm slice thickness. MDCT served as the reference standard, with 100% sensitivity by definition within this study design.

Magnetic resonance imaging protocol

All MRI examinations were performed on the 3.0T Siemens Skyra in the supine position. The following five non-contrast sequences were acquired: (1) T2-weighted HASTE (half-Fourier acquisition single-shot turbo spin-echo); (2) T1-weighted VIBE (volumetric interpolated breath-hold examination); (3) T2-weighted TrueFISP (true fast imaging with steady-state free precession); (4) T2-weighted BLADE (periodically rotated overlapping parallel lines with enhanced reconstruction); and (5) Ep2d diffusion-weighted imaging (DWI). Breath-hold maneuvers were employed for T1-VIBE and T2-HASTE acquisitions. Respiratory gating was applied where breath-hold capacity was limited.

Image analysis

CT and MRI images were reviewed at dedicated workstations, displayed simultaneously in a dual-screen configuration. For each MDCT-confirmed nodule, MRI detectability was recorded as true positive (identified on MRI) or false negative (missed on MRI). The maximum transaxial diameter was measured using integrated digital calipers on the image affording the clearest lesion delineation. Nodule margins were classified according to the Fleischner Society’s standardized glossary for thoracic imaging: margins were designated as well-circumscribed (sharply defined, smooth interface with adjacent lung parenchyma) or spiculated/irregular (linear strands radiating from the nodule margin into the surrounding lung or an ill-defined, non-smooth border) [2]. MRI signal intensity was categorized on T1- and T2-weighted sequences as hyperintense, isointense, or heterogeneous relative to the signal of the ipsilateral chest wall skeletal musculature, consistent with previously established MRI characterization criteria for pulmonary lesions [15].

Statistical analysis

Data were entered into SPSS Statistics, Version 21.0 (IBM Corp., Armonk, NY, USA), and Microsoft Excel 2019 (Microsoft Corp., Redmond, WA, USA). Continuous variables are expressed as mean ± standard deviation (SD) and categorical variables as frequencies and percentages. Because only patients with confirmed MDCT-detected nodules were enrolled, true-negative and false-positive classifications were not applicable; therefore, specificity, positive predictive value, and negative predictive value could not be calculated. Overall and size-stratified sensitivities of MRI were computed as the proportion of MDCT-detected nodules identified on the MRI. Agreement between CT- and MRI-derived maximum diameter measurements was assessed by Bland-Altman analysis [16]. The mean bias (systematic difference between CT and MRI measurements) and 95% limits of agreement (mean bias ± 1.96 SD of the differences) were calculated. Narrow limits of agreement and a bias approaching zero were considered indicative of clinically acceptable measurement concordance. For all inferential comparisons, a two-tailed p-value <0.05 was considered statistically significant.

## Results

Demographic and clinical profile

A total of 60 patients completed both MDCT and 3T MRI. The mean age was 49.67 ± 17.80 years (range = 18-85 years). Females slightly outnumbered males (female:male ratio = 1.14:1). Cough was the predominant presenting complaint, and most patients had experienced symptoms for one to three weeks before presentation. Detailed demographic and clinical characteristics of all participants are provided in Table 1.

**Table 1:** Baseline characteristics of study participants (N = 60). SOB = shortness of breath; SD = standard deviation

Characteristic	Category	n	%
Age (years)	Mean ± SD	49.67 ± 17.80	Range = 18–85
Sex	Female	32	53.3
	Male	28	46.7
Chief complaint	Cough	17	28.4
	Chest pain	13	21.6
	Fever with cough	13	21.6
	SOB	9	15.0
	Dyspnea with constitutional symptoms (weakness, weight loss)	5	8.4
	Hemoptysis with cough	3	5.0
Duration of symptoms	<1 week	17	28.3
	1–3 weeks	29	48.3
	>3 weeks	14	23.3

Nodule detection: overall and size-stratified sensitivity

MDCT identified 108 pulmonary nodules and masses across the 60 patients. MRI detected 94 of these (overall sensitivity = 87%). The largest nodule missed by MRI measured 7 mm. All lesions exceeding 7 mm were detected with 100% sensitivity. Table 2 summarizes detection rates stratified by lesion size.

**Table 2:** Size-stratified detection rate of MRI compared with MDCT. MDCT served as the reference standard (sensitivity = 100%). The 4–5 mm row initially recorded 5 MRI-detected nodules; verification against source data confirmed the correct value is 6, yielding a sensitivity of 75.0%. Percentages for MDCT and MRI columns are expressed as proportions of total MDCT-detected nodules (n = 108). Re-analysis using Fleischner Society 2017 categories: sensitivity <6 mm = 48.7% (19/39); 6–8 mm ≈ 95%; >8 mm = 100% (45/45). MDCT = multidetector computed tomography; MRI = magnetic resonance imaging

Nodule size	Nodules on MDCT, n (%)	Nodules on MRI, n (%)	Sensitivity (%)
<4 mm	11 (10.2%)	1 (0.9%)	9.0
4–5 mm	8 (7.4%)	6 (5.6%)	75.0
>5–7 mm	21 (19.4%)	20 (18.5%)	95.2
>7–10 mm	23 (21.3%)	23 (21.3%)	100.0
>10 mm	45 (41.7%)	45 (41.7%)	100.0
All nodules	108 (100%)	94 (87.0%)	87.0

Size measurement agreement

Bland-Altman analysis of the 94 concordantly detected nodules compared maximum transaxial diameters measured on CT against those measured on MRI. The mean bias was 0.65 mm (CT minus MRI), indicating a small but consistent tendency for MRI to underestimate nodule size relative to CT. The 95% limits of agreement ranged asymmetrically from −1.5 mm to +2.5 mm, representing a total span of 4.0 mm. This asymmetry reflects that MRI is more likely to underestimate than overestimate the diameter. The outer boundary of +2.5 mm approaches but does not exceed the conventional 2 mm growth threshold used in nodule surveillance, supporting adequate measurement concordance for clinical decision-making.

Nodule location and morphology

The right upper lobe was the most frequently involved (n = 31, 28.7% of nodules on MDCT), followed by the left upper lobe apico-posterior segment (n = 14, 13.0%), right lower lobe superior segment (n = 10, 9.1%), and bilateral lower lobe posterior segments (n = 18, 16.7%). MRI correctly localized all detected nodules to the same anatomical segment identified on MDCT. In total, 49 (81.6%) patients had well-circumscribed nodules; 11 (18.4%) had spiculated or irregular margins, which appeared less sharply defined on MRI due to motion and susceptibility artifacts (Table 3). Concordance for cavitation (n = 10, 16.7%), pleural effusion (n = 19, 31.7%; comprising unilateral right-sided effusion in seven cases, unilateral left-sided effusion in six cases, and bilateral effusion in six cases), and mediastinal lymphadenopathy (n = 14, 23.3%) between the two modalities was 100% (Table 3).

**Table 3:** Concordance of morphological findings between MDCT and MRI. Concordance assessed among the 94 nodules detected on both modalities. MDCT = multidetector computed tomography; MRI = magnetic resonance imaging

Feature	MDCT, n (%)	MRI, n (%)	Concordance
Well-circumscribed margins	49 (81.7%)	49 (81.7%)	100%
Spiculated/Irregular margins	11 (18.3%)	11 (18.3%)	100% detected
Cavitation present	10 (16.7%)	10 (16.7%)	100%
Cavitation absent	50 (83.3%)	50 (83.3%)	100%
Pleural effusion (any)	19 (31.7%)	19 (31.7%)	100%
No pleural effusion	41 (68.3%)	41 (68.3%)	100%
Mediastinal lymphadenopathy	14 (23.3%)	14 (23.3%)	100%

Representative case illustrations

**Figure 3:**
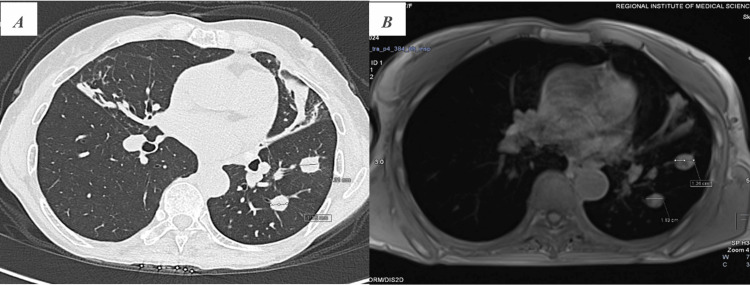
(A) Axial view of HRCT of the thorax showing well-defined pulmonary nodules in the LLL anteromedial and posterior segments. (B) T1-VIBE MRI sequence; corresponding nodules appear hyperintense in the same LLL segments, demonstrating reliable localization. Note: not all nodules visible on CT (Panel A) are identifiable on MRI (Panel B), consistent with the size-dependent detection limitation of MRI for sub-5 mm lesions. Right lung findings were separately evaluated in additional cases. HRCT = high-resolution computed tomography; LLL = left lower lobe; T1-VIBE = volumetric interpolated breath-hold examination; MRI = magnetic resonance imaging

**Figure 4:**
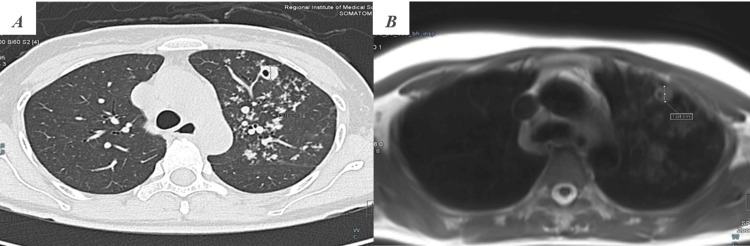
(A) Axial view of HRCT of the thorax; well-defined pulmonary nodule with central cavitation in the LUL anterior segment. (B) Corresponding T1-VIBE MRI sequence; the same nodule appears hyperintense with a visible cavitary component, concordant with CT. Note: the pleural tag visible on CT (Panel A) is not clearly reproduced on MRI (Panel B), illustrating reduced MRI sensitivity for fine structural nodule-pleura interface details. HRCT = high-resolution computed tomography; LUL = left upper lobe; T1-VIBE = volumetric interpolated breath-hold examination; MRI = magnetic resonance imaging

**Figure 5:**
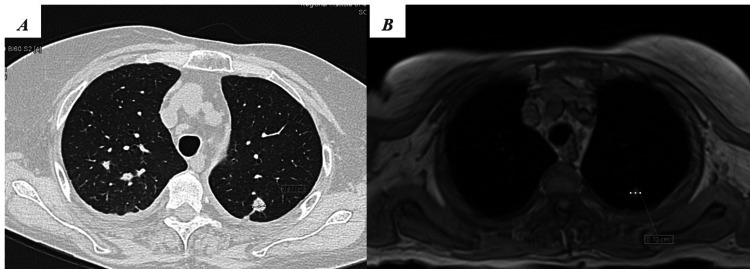
(A) Axial view of HRCT of the thorax demonstrating a well-defined pulmonary nodule in the LUL apico-posterior segment. (B) T1-VIBE MRI sequence; the nodule is clearly depicted as hyperintense in the same segment, confirming high sensitivity for this size range (>7 mm). HRCT = high-resolution computed tomography; LUL = left upper lobe; T1-VIBE = volumetric interpolated breath-hold examination; MRI = magnetic resonance imaging

Figures 3-5 present representative CT-MRI paired images from the study cohort. Each pair demonstrates concordant nodule detection across different anatomical locations and morphological appearances. These illustrative cases span the size range in which MRI performs reliably (>5 mm) and document typical MRI signal characteristics (hyperintense or heterogeneous on T2-weighted sequences).

Magnetic resonance imaging signal characteristics

Among the 94 nodules detected on MRI, 47 (50.0%) were hyperintense on both T1- and T2-weighted sequences, 37 (39.4%) showed heterogeneous signal intensity, and 10 (10.6%) were isointense relative to skeletal muscle. The hyperintense pattern on T2-weighted imaging, well illustrated in the paired figures above, was the predominant appearance and reflected the solid or mixed-density character of nodules confirmed on CT. A heterogeneous signal was predominantly observed in larger, partly necrotic masses.

## Discussion

This study evaluated the diagnostic performance of a multi-sequence 3.0T MRI protocol for detecting pulmonary nodules and masses, yielding an overall sensitivity of 87% against MDCT as the reference standard, with 100% sensitivity for lesions exceeding 7 mm. These figures situate our findings within a broader and growing body of evidence supporting pulmonary MRI as a clinically viable, radiation-free alternative to CT for nodule surveillance. Cieszanowski et al. reported 100% sensitivity for nodules >8 mm at 1.5T, with declining performance for smaller lesions [14]. Sanchez et al. similarly found superior MRI performance for nodules >4 mm versus smaller lesions on a 3T platform using UTE, T1 gradient-echo, and HASTE sequences [17]. Hinsen et al., using low-field MRI, reported detection accuracy of 100% for nodules >6 mm and 80% for 4-6 mm [18], and Periaswamy et al. [19] reported 87.96% using UTE MRI at 3T in an oncology cohort. By contrast, Liu et al., in a meta-analysis of eight studies encompassing 653 patients, concluded that CT retains superior sensitivity over MRI overall, although both modalities demonstrated high diagnostic accuracy [8]. The present study aligns with the more optimistic end of this spectrum, likely reflecting the advantage of 3T field strength and the use of five complementary sequences rather than a single acquisition. This protocol design has consistently been shown to improve detection rates compared with single-sequence MRI [13,17].

The size-stratified sensitivity profile, ranging from 9% for sub-4 mm lesions to 100% for nodules exceeding 7 mm, is consistent with findings across multiple prior studies regardless of field strength. Cieszanowski et al. reported 100% sensitivity for nodules >8 mm, 87.5% for 6-8 mm, and 75% for 4-6 mm at 1.5T [14], closely mirroring our respective values of 100%, 95.2%, and 75% at 3T. Feng et al., also using 3T MRI, reported a 100% detection rate for nodules >8 mm and significantly lower rates for subsolid and smaller nodules, consistent with our observation that sub-5 mm lesions remain the principal limitation of pulmonary MRI [6]. Schroeder et al. reported sensitivities of 73%, 86.3%, 95.7%, and 100% for lesions <1 <3 mm, 3-5 mm, 6-10 mm, and >10 mm, respectively, using HASTE at 1.5T [12], a gradient remarkably similar to the present study, confirming that spatial resolution relative to lesion size, rather than field strength alone, is the primary determinant of MRI detectability. Vogt et al. similarly demonstrated that HASTE MRI sensitivity increased from 94.9% for 5-10 mm nodules to 100% for lesions exceeding 3 cm [10], further corroborating this size-dependent relationship. Notably, Regier et al. [20], using a high-field 3T system with porcine lung explants, similarly identified critical sequence-dependent detection thresholds for sub-centimeter nodules, confirming that these limits are intrinsic to pulmonary MRI physics rather than artifacts of specific study design.

It is important to acknowledge that the size categories employed in the present study (<4 mm, 4-5 mm, >5-7 mm, >7-10 mm, >10 mm) were selected to align with published MRI detection literature rather than with the Fleischner Society 2017 management thresholds (<6 mm, 6-8 mm, >8 mm) [2]. When the present data are re-examined using Fleischner-aligned categories, MRI sensitivity was 48.7% for nodules <6 mm (19 of 39 detected) and 100% for nodules >8 mm (45 of 45 detected). This re-analysis reinforces the clinically important conclusion that 3T MRI performs reliably within the >8 mm category that Fleischner guidelines identify as the highest-risk tier, justifying positron emission tomography-computed tomography (PET-CT) or tissue sampling, while remaining less reliable for the sub-6 mm tier that requires only periodic surveillance or no follow-up in low-risk individuals [2]. Future studies should adopt standardized Fleischner size strata to facilitate cross-study comparison.

The clinical relevance of sub-4 mm non-detection must be interpreted in context. Current Fleischner Society guidelines recommend no routine follow-up for nodules smaller than 6 mm in low-risk individuals [21], and at least 99% of nodules measuring less than 4 mm are benign [22]. Sommer et al., in a high-risk screening population, reported an overall MRI sensitivity of only 48% but a significantly higher sensitivity of 78% specifically for malignant nodules [11], suggesting that MRI is inherently better suited to detecting the lesions most likely to be clinically consequential. This observation supports the notion that MRI’s reduced sensitivity for sub-centimeter lesions may carry less clinical weight than its statistical magnitude implies.

Regarding size measurement accuracy, our Bland-Altman analysis demonstrated a mean bias of 0.65 mm and limits of agreement of -1.5 to +2.5 mm, indicating excellent concordance between CT and MRI measurements. These results are directly comparable to those of Darcot et al. [23], who reported similarly narrow limits of agreement in a prospective CT-MRI comparison, and to Cieszanowski et al. [14], who found no statistically significant difference in nodule diameter measurements between the two modalities for detected lesions. Heye et al. also confirmed that MRI size measurements strongly correlated with CT for lesions exceeding 1 cm [13]. Collectively, these data, including the present study, establish that MRI provides measurement fidelity sufficient for clinical decision-making in nodule surveillance, where a 2 mm diameter change conventionally denotes meaningful growth.

Among the 94 nodules and associated thoracic findings detected on both modalities, 3T MRI demonstrated 100% concordance with MDCT for cavitation, pleural effusion, and mediastinal lymphadenopathy. This is consistent with the well-established superiority of MRI for soft-tissue characterization and mediastinal assessment. Usuda et al. demonstrated that MRI, particularly DWI and T2-weighted sequences, outperformed FDG-PET/CT in differentiating benign from malignant pulmonary nodules and masses [24], underscoring MRI’s potential not merely as a nodule detection tool but as a comprehensive thoracic staging modality. Yi et al. similarly confirmed that 3T MRI depicted clinically significant 5-10 mm non-calcified nodules at rates comparable to CT on both T1 and T2 sequences, while also providing superior soft-tissue contrast for mediastinal evaluation [25].

The upper lobe predominance of nodules in our cohort, particularly the right upper lobe apical and left upper lobe apico-posterior segments, reflects the well-known predilection of post-primary tuberculosis for these regions, which is the dominant etiology of pulmonary nodules in northeast India. This contrasts with Western screening cohorts, where adenocarcinoma precursors and ground-glass nodules constitute a larger proportion of detected lesions. Notably, Feng et al. similarly reported upper lobe predominance in their Chinese cohort [6], where tuberculosis and its sequelae also contribute substantially to the nodule burden. Despite this difference from Western datasets, the technical performance of MRI for solid nodule detection is expected to be etiology-independent, as the imaging challenge, detecting a soft-tissue opacity against an air-filled background, is uniform across all nodule types. Whether anatomical lobar or segmental position independently influenced MRI nodule detectability was not systematically evaluated in the present study; lower lobe nodules, in particular, may be disproportionately affected by cardiac pulsation and diaphragmatic motion artifacts on non-breath-hold sequences, an effect that future studies should formally quantify.

Margin characterization in the present study was reliable for well-circumscribed nodules but was qualitatively less precise for spiculated margins, particularly in the presence of respiratory motion artifact. This limitation is well recognized in the literature. Cieszanowski et al. similarly noted that MRI margin definition was inferior to CT for smaller and irregularly marginated lesions [14]. The implementation of UTE sequences, which substantially reduce susceptibility artifacts at tissue-air interfaces, has been shown by Bonert et al. [26] and Burris et al. [27] to improve both detection rates and morphological detail for small nodules and represents a logical next step in protocol optimization for future studies at this institution. Furthermore, whether spiculated or irregular margin morphology contributed to systematic size underestimation on MRI, with potential implications for follow-up decisions where nodule size relative to a threshold determines the imaging interval, was not formally assessed and represents an important area for prospective investigation.

The radiation dose benefit of MRI-based surveillance is particularly relevant in the Indian clinical context, where many patients with pulmonary nodules attributable to tuberculosis, sarcoidosis, or other benign granulomatous diseases require prolonged follow-up over years to decades. A single high-resolution CT delivers approximately 6-8 mSv, and cumulative doses from repeated examinations may reach tens of millisieverts, carrying a measurable radiation-attributable cancer risk [4]. Meier-Schroers et al. [28] demonstrated that MRI-based Lung-RADS scoring correlated significantly with low-dose CT across two screening rounds and never underestimated high-risk categories, confirming that MRI can replicate CT-equivalent risk stratification without radiation exposure. For patients in this epidemiological setting, periodic substitution of MRI for CT at surveillance intervals is both radiologically justified and practically beneficial.

It must equally be acknowledged, however, that the 0.85% radiation-attributable cancer risk from annual CT screening [4] must be weighed against the competing risk of delayed malignancy detection in nodules <8 mm, where MRI sensitivity remains suboptimal. The clinical decision to substitute MRI for CT at any surveillance interval should therefore be individualized, taking into account the patient’s age, baseline cancer risk, nodule morphology, and whether the lesion falls within the size range where MRI has demonstrated adequate performance in this and prior studies.

Several limitations must be acknowledged. The restriction of enrollment to MDCT-confirmed nodule cases precluded calculation of specificity and predictive values. Absence of histopathological confirmation for most lesions prevented assessment of MRI performance against tissue diagnosis. The sample size, though adequate by power calculation, limits the precision of size-stratified estimates, particularly for the sub-5 mm strata. Individual MRI sequence performance was not separately benchmarked, and the study was conducted at a single center, which may limit generalizability. Critically, the present cohort comprised predominantly solid pulmonary nodules; ground-glass opacity nodules and semisolid (part-solid) nodules were not separately characterized or reported. Given that ground-glass opacity and semisolid lesions exhibit fundamentally different MRI signal characteristics compared with solid nodules and represent an increasingly important category in lung cancer screening programs, the performance of 3T MRI for these subtypes cannot be extrapolated from the present data and warrants dedicated investigation. Similarly, the effects of overlying pathology (e.g., consolidation, pleural effusion), lobar position, and spiculated margin morphology on MRI-derived size accuracy were not formally assessed and merit future study. Future multicenter prospective studies with histopathological correlation, larger cohorts, and systematic individual sequence analysis, including UTE and contrast-enhanced VIBE protocols, would help consolidate the evidence base for 3T MRI in structured pulmonary nodule surveillance programs.

## Conclusions

A multi-sequence 3.0T MRI protocol demonstrated high sensitivity for pulmonary nodules and masses, achieving 100% detection for lesions exceeding 7 mm with excellent size-measurement concordance with MDCT (mean bias = 0.65 mm). Secondary thoracic findings, including cavitation, pleural effusion, and mediastinal lymphadenopathy, were concordantly identified. Representative CT-MRI image pairs confirm reliable nodule depiction across diverse anatomical locations and morphological patterns. These results support the role of 3T MRI as a clinically viable, radiation-free alternative to MDCT for surveillance of known solid pulmonary nodules exceeding 7 mm in appropriate clinical contexts, particularly for patients requiring repeated long-term imaging. Prospective multicenter studies with histopathological correlation, standardized Fleischner Society size strata, and systematic individual sequence benchmarking would help consolidate the evidence base and delineate the optimal role of 3T MRI within structured pulmonary nodule surveillance programs.
